# RETRACTION: Ventral Hernia Repair in Conjunction With LIPO‐ABDOMINOPLASTY in Overweight Patients: A Comprehensive Approach

**DOI:** 10.1111/iwj.70598

**Published:** 2025-04-21

## Abstract

**RETRACTION:** ErfanM. A. , NasserS. , El WardanyI. , and ZaazouM. M. T. , “Ventral Hernia Repair in Conjunction With LIPO‐ABDOMINOPLASTY in Overweight Patients: A Comprehensive Approach,” International Wound Journal 20, no. 5 (2023): 1558–1565, 10.1111/iwj.14011.36695339 PMC10088855

The above article, published online on 25 January 2023, in Wiley Online Library (http://onlinelibrary.wiley.com/), has been retracted by agreement between the journal Editor in Chief, Professor Keith Harding; and John Wiley & Sons Ltd. Following an investigation by the publisher, all parties have concluded that this article was accepted solely on the basis of a compromised peer review process. In addition, the investigation found that the authors provided incomplete ethical approval information for the study. The editors have therefore decided to retract the article. The authors did not respond to our notice regarding the retraction.

**RETRACTION:** M. A. Erfan , S. Nasser , I. El Wardany , and M. M. T. Zaazou , “Ventral Hernia Repair in Conjunction With LIPO‐ABDOMINOPLASTY in Overweight Patients: A Comprehensive Approach,” International Wound Journal 20, no. 5 (2023): 1558–1565, 10.1111/iwj.14011.36695339 PMC10088855

The above article, published online on 25 January 2023, in Wiley Online Library (http://onlinelibrary.wiley.com/), has been retracted by agreement between the journal Editor in Chief, Professor Keith Harding; and John Wiley & Sons Ltd. Following an investigation by the publisher, all parties have concluded that this article was accepted solely on the basis of a compromised peer review process. In addition, the investigation found that the authors provided incomplete ethical approval information for the study. The editors have therefore decided to retract the article. The authors did not respond to our notice regarding the retraction.

